# The timing of elective caesarean delivery between 2000 and 2009 in England

**DOI:** 10.1186/1471-2393-11-43

**Published:** 2011-06-08

**Authors:** Ipek Gurol-Urganci, David A Cromwell, Leroy C Edozien, Chidimma Onwere, Tahir A Mahmood, Jan H van der Meulen

**Affiliations:** 1 Department of Health Services Research and Policy, London School of Hygiene and Tropical Medicine, London, UK; 2 Office of Research and Clinical Audit, Lindsay Stewart R&D Unit, Royal College of Obstetricians and Gynaecologists, London, UK; 3 Maternal and Fetal Health Research Centre, St Mary's Hospital, Manchester, UK

## Abstract

**Background:**

In 2004, the National Institute for Clinical Excellence (NICE) recommended that an elective caesarean section for an uncomplicated pregnancy should not be carried out before 39 completed weeks due to increased risk of respiratory morbidity in newborns. We describe the trends and variation across 63 English NHS trusts in the timing of elective caesarean section (CS) for low-risk singleton deliveries.

**Methods:**

We identified elective CS deliveries between 1^st ^April 2000 and 28^th ^February 2009 in English NHS trusts using the Hospital Episode Statistics. We selected women with uncomplicated pregnancies who had an elective CS delivery after 34 completed weeks of gestation, and analysed the trends and the trust-level variation in the timing of elective CS. The impact of the NICE guidance on the monthly rate of elective CS deliveries performed after 39 weeks was estimated using an interrupted time-series design with autoregressive integrated moving average (ARIMA).

**Results:**

There were 118,456 elective CS deliveries at the 63 NHS trusts. The overall proportion of elective CS deliveries done after 39 completed weeks steadily increased from 39% in 2000/01 to 63% in 2008/09. The proportions rose from 43% to 67% for women with breech presentation and from 35% to 62% for women with a previous CS. There was significant variation across NHS trusts in each year; in 2008/09, with the proportions of elective CS done after 39 weeks ranging from 28% to 89% (Inter-quartile range limits: 54% to 72%). We found a small but statistically significant increase in the proportion immediately after the publication of the NICE guidance, but its rate of growth rate declined slightly thereafter.

**Conclusions:**

NHS trusts in our study have responded to the new evidence on the benefits of delaying elective CS to after 39 weeks gestation. However, substantial differences between NHS trusts remain, which indicates there is room for further improvement. We suggest that maternity services and commissioners adopt the "timing of elective caesarean" as a quality indicator to support clinical practice.

## Background

Since the mid-1990s, various studies have reported that elective caesarean sections (CS) performed before 39 completed weeks of gestation are associated with an increased likelihood of respiratory morbidity in newborns and admissions in neonatal intensive care [1-5], which has considerable economic costs as well as psychological costs of separating mother and baby right after birth. The incidence of respiratory morbidity has been estimated to be 7.4 to 11.4% for elective CS deliveries at 37 weeks gestation, 4.2 to 8.4% at 38 weeks gestation and 0.8 to 2.1% for 39 weeks [3,6-8].

In 2004, the National Institute of Clinical Excellence (NICE) *Clinical Guideline: Caesarean Section *recommended that planned caesarean section should not be routinely carried out before 39 completed weeks of gestation [9]. Similar recommendations have been included in guidance from other countries [10] and recent publications have provided further evidence on the relationship between the timing of an elective caesarean section and neonatal respiratory illness [6,7,11-14].

Elective CS rates have been increasing in England, rising from 5% of all births in 1990 to 10% in 2008 [15]. Some of this increase reflects changes in clinical practice, with most women with breech presentation now being delivered by an elective CS [16]. Furthermore, there are an increasing number of women with a previous CS, and a greater proportion of these are opting for another elective CS in preference to a trial of vaginal delivery. Finally, an increase in maternal requests for elective CS delivery has also been reported [9].

To date, there have been no published studies analysing the response of maternity units in England to the growing evidence-base on the timing of elective CS and the guidance issued by NICE. Poor compliance with the guidance could increase the number of babies at risk of avoidable respiratory morbidity.

In this paper, we describe the trends among English NHS acute trusts in the timing of elective CS for low-risk women delivering singletons after 34 completed weeks of gestation between 2000 and 2009. We also assess the extent of variation between individual NHS trusts regarding the practice of delaying elective CS to after 39 completed weeks and examine whether there was a measurable change in clinical practice after the publication of the NICE guideline in 2004.

## Methods

### Population selection

We used data extracted from the routinely collected Hospital Episode Statistics (HES) database, which captures patient demographics and clinical information for all admissions to English NHS trusts. We included all singleton elective CS delivery episodes in NHS trusts collecting gestation data, from 1^st ^April 2000 to 28^th ^February 2009. As the completeness of gestation information varied between NHS trusts, we restricted the analysis to trusts that had gestation data in more than 50% of the delivery episodes in at least seven of the nine years covered in the study. Women were allocated to the NHS trusts that existed in February 2009 to take account of previous mergers.

The HES core fields contains diagnostic information coded using the International Classification of Diseases (ICD), 10th revision, and operative procedures described using the UK Office for Population Censuses and Surveys classification (OPCS), 4th revision. Gestational age is recorded in the maternity fields in HES and is defined as the number of completed weeks of gestation. We use the same convention in the text below. For example, at 37 weeks implies gestations of 37 weeks + 0 days, before 34 weeks implies gestations ≤ 33 weeks + 6 days, and after 39 weeks implies gestations ≥ 39 weeks + 0 days.

Women were included in this study if their HES record contained the OPCS code R17 (elective caesarean section) in any of the core operative procedure fields. We excluded women who had their elective CS before 34 weeks of gestation or had a condition coded in any diagnosis field that could be a contra-indication for delaying an elective CS. These risk factors included pre-existing and gestational diabetes (ICD10 codes: E10, E11, O24), hypertensive disorders including pre-eclampsia and eclampsia (O10-O11, O13-O16), premature rupture of membranes (O42), polyhydramnios (O40), oligohydramnios (O41.0), excessive or poor fetal growth (O36.5, O36.6), and placenta praevia (O44).

### Analysis

For low-risk women who deliver singletons after 34 weeks, we initially analysed the proportion of all elective CS deliveries that were performed after 37, 38, 39 and 40 weeks of gestation. Deliveries were grouped into monthly periods to generate the time-series. For elective CS deliveries after 39 weeks, we repeated the time trend analysis for each of the three groups: women who had a pregnancy with a breech presentation (ICD10 codes: O31.1, O64.1, O80.1, O83.0, or O83.1), those who had a previous caesarean section/uterine scar (ICD10 code: O34.2) and those without these two CS indications.

The impact of the NICE guidance on the proportion of elective CS performed after 39 weeks gestation was analysed using an interrupted time series design with autoregressive integrated moving average (ARIMA) [17,18]. The model was applied to data aggregated on a monthly basis and contained terms to detect a shift in the average at the time of publication and a change in the rate of growth. The numbers of time points before and after the publication of NICE guidance in April 2004 were 48 and 59 respectively.

Finally, we examined the proportion of elective CS performed after 39 weeks at the individual trusts. The data were grouped by financial year (e.g. April 2000 to March 2001), and the average change in the annual proportions was estimated using linear regression. All analysis was performed in STATA version 10. The time-series are presented in three-month intervals rather than monthly for clarity of presentation.

Under UK National Research Ethics Service guidance, this study constituted service evaluation and did not require ethics approval because it involved the analysis of existing, anonymised data with the primary aim of describing variations in practice.

## Results

Among the 140 NHS trusts at which women were delivered by elective CS, 63 English NHS trusts provided adequate gestation information to fulfil our inclusion criteria. In these trusts, there were 145,492 singleton elective CS episodes between April 2000 and February 2009, corresponding to 33% of all elective CS deliveries in England (n = 439,415). Among these episodes, 27,036 (18.6%) women delivered before 34 weeks gestation or had a contra-indication for delaying an elective CS delivery, and were excluded.

The total number of deliveries included in the analysis was 118,456. Of these, 25,521 (22%) women had a breech presentation, 62,012 (52%) women had a previous CS, while 30,923 (26%) women had neither indication. The number of CS deliveries increased by 46% from 9,942 in 2000/01 to 14,498 in 2008/09. The number of women with previous CS increased from 4,974 to 8,318, constituting 57% of all CS deliveries in 2008/09 as compared with 50% in 2000/01.

**Figure 1:**
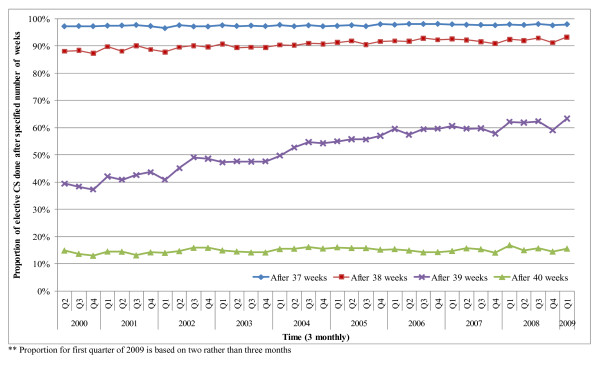
**Trends in the timing of elective caesarean section deliveries**.

The proportion of elective CS deliveries performed after 37, 38, 39 and 40 weeks over time is shown in Figure 1. The proportion of elective CS performed after 37 weeks remained steady at around 97% of all elective CS; the proportion performed after 40 weeks also remained steady at around 15%. In contrast, the proportion performed between 39 and 40 weeks increased from 39% to 63% between April 2000 and February 2009, while the proportion of elective CS deliveries done between the 37^th ^and 39^th ^week of pregnancy decreased from 49% to 30%.

**Figure 2:**
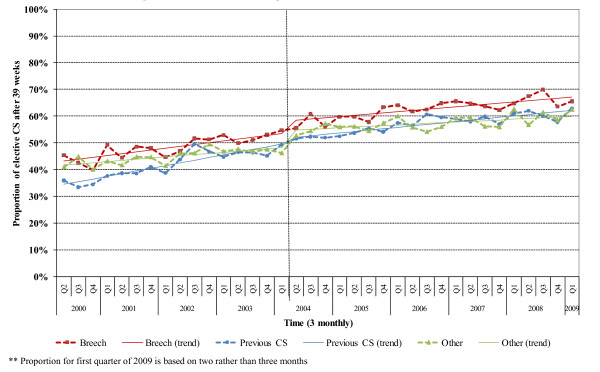
**Trends in the rate of elective caesarean sections delayed performed after 39 weeks, by the two main indications for elective caesarean section**. Fitted line shows the predicted effect of the NICE guideline on the individual rates.

Similar changes in the timing of elective CS occurred for women with different indications. The proportion of women with breech presentation being delivered after 39 weeks rose steadily from 43% to 67% between April 2000 and February 2009. A similar trend occurred among women with previous CS, increasing from 35% to 62% (Figure 2). After the publication of the NICE guidance in April 2004, there was an immediate increase in the proportion of elective CS performed after 39 weeks for all women (3.5%, p = 0.001), for women with breech presentation (4.4%, p = 0.002) and for women with neither indication (6.3%, p <0.001). The shift in the proportion for women with previous CS was not statistically significant. The growth rate of the proportion in the post NICE publication period decreased for all women (p = 0.004) and for those women with previous caesarean section (p = 0.001).

**Figure 3:**
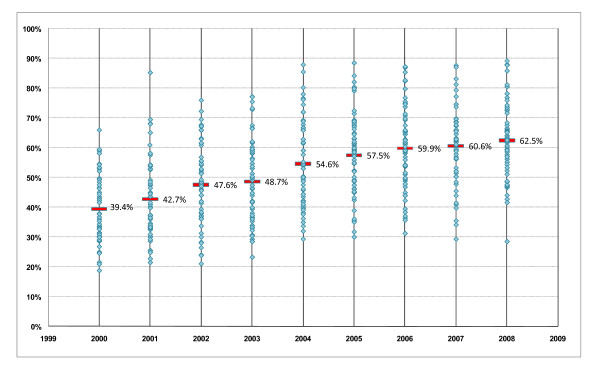
**Proportion of rate of elective caesarean sections performed after 39 weeks in NHS trusts between April 2000 and February 2009, by financial year**. Overall proportion for all women is shown as bars (and values) for comparison.

Figure 3 shows the proportions of elective CS performed after 39 weeks at individual NHS trusts by financial year. The increasing median shows a general improvement in the practice of delaying elective CS overall. In 2000/01, only one trust performed more than 60% of their elective CS procedures after 39 weeks gestation; in 2008/09, more than half of the trusts exceeded this level. However, the variation between trusts did not decrease over time. The inter-quartile range in 2000/01 was 17.5% (limits: 31.1% to 48.6%) and was similar in each subsequent year. The inter-quartile range in 2008/09 was 17.6% (limits: 54.9% to 72.5%). Moreover, the changes over time at each NHS trust were not similar. There were 14 trusts (22%) at which the proportion of elective CS performed after 39 weeks changed by less than 1% per year, while at another 18 trusts (29%), it increased by an average of 4% per year.

## Discussion

Since 2000, the practice of delaying elective CS has been increasingly adopted by English NHS trusts. In low-risk women who deliver singletons after 34 weeks, 63% of elective CS were performed after 39 weeks in the first quarter of 2009 at the 63 NHS trusts included in this analysis. This suggests that NHS trusts have responded to the evidence on the risk of respiratory distress syndrome that is associated with elective CS before 39 weeks gestation [1-5]. The publication of the NICE guidance may have contributed to this but its effect is unclear. There was a small but statistically significant increase in the proportion of elective CS performed after 39 weeks with the publication of the NICE guidance, but the rate of growth declined slightly thereafter.

The proportions of elective CS performed after 39 weeks at individual NHS trusts differed markedly and the variation has not decreased over time. While a quarter of the 63 NHS trusts performed over 72% of elective CS after 39 weeks, the proportion was under 50% at 11 trusts (17%). It is not clear what proportion of low-risk women with singleton pregnancies could have their elective CS safely delayed to after 39 weeks but the variation between NHS trusts suggests some trusts could increase the proportion further.

### Methodological strengths and limitations

Strength of our study comes from its inclusion of multiple NHS trusts and the large number of elective CS deliveries. The sample included small (<2500 deliveries), medium (2500-4000) and large (>4000 deliveries) NHS trusts and the trusts were spread across all English regions ('Strategic Health Authorities'). The characteristics of the women in the included and excluded NHS trusts were also similar: mean age (31.4 years in both groups), the proportion of women identified as low risk (71.9% vs. 72.4%), breech presentation (20.3% vs. 19.6%) and previous CS (51.0% vs. 50.0%).

In this analysis, method of delivery was identified using 3-digit OPCS codes and using these broader categories has been shown to be more reliable than using the more specific 4-digit codes [19]. The coding of elective CS in administrative databases like HES has also been reported as accurate (Kappa = 0.88 for elective CS) [20]. Consequently, errors in the coding of the mode of delivery are unlikely to account for the observed trends or variation between NHS trusts.

We limited the analysis to uncomplicated pregnancies to minimise the influence of conditions that might necessitate intervention before 39 weeks gestation. However, incomplete diagnostic coding of obstetric conditions could have inflated the number of elective CS performed between 35^th ^and 39^th ^weeks and so have led to the proportion of elective CS performed after 39 weeks being underestimated. Changes in coding over time could have influenced our results but any change would have affected the proportion of elective CS deliveries at each week of gestation. That we observed a change principally in elective CS after 39 weeks suggests the effect of coding changes on the results is small. Differences between NHS trusts in the degree to which factors precluding delay were recorded would have contributed to the variation observed between trusts. However, the effect of this is likely to be small in comparison to the level of variation observed and the prevalence of HES-derived indications was similar to those reported by the National Sentinel Caesarean Section Audit (NSCSA) [21].

Over the past twenty years, the obstetricians have increasingly used early pregnancy ultra-sound scanning to determine the expected due date of delivery. Our study has assessed clinicians' practice regarding delivery after 39 weeks based on their own estimate of gestational age for delivery. Consequently, these differences are unlikely to account for the observed trends in practice nor the variation observed between NHS trusts.

Our analysis of the impact of NICE guidance on practice is limited because we could not control for other factors that may have influenced practice during the study period. However, our data covered five years after the publication of the guidance, which is a sufficient time interval for its dissemination and implementation. We feel it is reasonable to conclude that, in the sample of NHS trusts examined, the NICE guideline had only a small effect on clinical practice.

### Implications on policy and practice

The timing of elective CS is influenced by several social and medical indications. Women whose operation is planned for after 39 weeks may present in labour or with increased maternal or fetal risks such as a sudden rise in blood pressure or ante partum haemorrhage at 38^th ^or 39^th ^week and have an emergency CS. This might be one reason why a higher proportion of women with breech presentations were delivered after 39 weeks of gestation compared to women with a previous CS. Although the NICE guidance does not make the distinction for the timing of elective CS for women with breech versus previous CS indications, some obstetricians might conclude that, due to various fetal and maternal risks, 39^th ^week is the optimal time for an elective CS in women with a previous CS.

It is possible that, in smaller maternity units with a limited number of obstetricians and anaesthetists, some elective CS are performed before 39 weeks gestation to reduce the risk of women going into labour and requiring an emergency CS when staff coverage is lower. This is unlikely to be the principal reason for the differences between NHS trusts observed. In our study, the proportion of elective CS done after 39 weeks was only slightly less on weekends than weekdays (57.8% vs. 61.6% in 2008/09). Moreover, in small NHS trusts (with less than 2500 births), the proportion of elective CS done after 39 weeks was higher than the proportion in medium/large hospitals (66.9% vs. 60.8% in 2008/09).

To date, the HES database has not permitted the delivery record of the mother to be linked to the baby record, and consequently, it was not possible to determine how the change in the pattern of CS timing influenced neonatal respiratory morbidity. With due caution, it is possible to estimate the reduction in the number of newborns at risk of respiratory distress by combining the observed number of elective CS at particular gestational ages with their associated incidence rates of respiratory morbidity. From published studies [3,6-8], we took conservative incidence rates of 18%, 7%, 4% and 1% for preterm, 37, 38 and 39 weeks gestation respectively. Combining these with the observed timings in the 63 NHS trusts suggests that the proportion of newborns who developed respiratory morbidity changed from 3.3% of the 9,942 elective CS deliveries in 2000/01 to 2.5% of the 14,498 elective CS in 2008/09. While the reduction in proportion of newborns with respiratory morbidity following the improved practice of delaying CS is significant, the estimated number of neonates that require special care baby unit beds has slightly increased from 331 to 360 due to the increase in the number of elective CS.

Decisions about the timing of an elective CS are becoming increasingly important to obstetricians because the demand for elective CS continues to grow [15]. There is evidence of considerable maternal morbidity due to repeat CS, including increased risks of haemorrhage, uterine rupture, and placenta praevia. Moreover, the benefits of delaying elective CS need to be balanced against the risks. These include a slightly higher risk of stillbirth [7] and the risk of spontaneous labour prior to the procedure. Morrison estimated that 10% of women planned to have an elective CS would go into labour if the procedure was performed at 39 weeks instead of 38 weeks [3]. Consequently, local quality improvement initiatives concerned with the timing of elective CS need to be considered within this wider context. While improvements in practice related to the timing of elective CS can avoid neonatal respiratory morbidity and reduce pressure on neonatal intensive care services, neonatal and maternal outcomes may be improved further if it is not considered in isolation.

Systematic reviews of guideline implementation strategies identify various strategies to increase the uptake of the recommendation by clinicians. These include dissemination of educational materials, educational meetings and local opinion leaders, although their success was highly dependent upon context [22,23]. A review focused on guideline implementation in obstetric care reported that educational strategies with medical providers were ineffective and recommended a multifaceted strategy incorporating audit and feedback and using local opinion leaders to change practice [24].

In the USA, organisations have begun using indicators to monitor the proportion of elective CS performed after 39 completed weeks on women with an uncomplicated pregnancy [25]. A recent paper has evaluated the effectiveness of three approaches to reduce elective early term delivery (defined as a planned delivery for women without a recognisable medical or obstetric indication for delivery) and concluded that a 95% rate of elective delivery after 39 weeks would be a reasonable national quality benchmark in the USA [26]. It is possible that the NHS may also adopt this indicator, although a target value of 95% is unlikely to be appropriate for the English NHS given that few trusts currently have values above 80%. However, the completeness of HES would need to improve before it could be used to derive this indicator for all NHS trusts in England. Work is ongoing to develop quality indicators to support quality accounts [27] and these indicators will also form the basis of "commissioning for quality and innovation" (CQUIN) [28]. Such a contractual arrangement could be a powerful incentive to change clinical practice.

## Conclusions

In conclusion, the results of this study suggest English NHS trusts have responded to the increasing evidence on the benefits of delaying elective CS, with nearly two thirds of procedures being performed after 39 weeks in the first months of 2009. Nonetheless, there were substantial differences between NHS trusts in the proportion of elective CS performed after 39 weeks, which indicates there is room for further improvement. Maternity services could use the "timing of elective caesarean" as a quality indicator to support local clinical audit and effort should be made to improve HES data so that it is possible to produce figures for all English NHS trusts.

## Competing interests

The authors declare that they have no competing interests.

## Authors' contributions

LCE, TAM, IGU, DC, and JvdM designed the study. IGU analysed the data supported by DC and JvdM. IGU wrote the first draft. All authors contributed to the interpretation of the results, revised further drafts, and approved the final manuscript.

## Pre-publication history

The pre-publication history for this paper can be accessed here:

http://www.biomedcentral.com/1471-2393/11/43/prepub
